# In vivo isotope tracing reveals a requirement for the electron transport chain in glucose and glutamine metabolism by tumors

**DOI:** 10.1126/sciadv.abn9550

**Published:** 2022-08-31

**Authors:** Panayotis Pachnis, Zheng Wu, Brandon Faubert, Alpaslan Tasdogan, Wen Gu, Spencer Shelton, Ashley Solmonson, Aparna D. Rao, Akash K. Kaushik, Thomas J. Rogers, Jessalyn M. Ubellacker, Collette A. LaVigne, Chendong Yang, Bookyung Ko, Vijayashree Ramesh, Jessica Sudderth, Lauren G. Zacharias, Misty S. Martin-Sandoval, Duyen Do, Thomas P. Mathews, Zhiyu Zhao, Prashant Mishra, Sean J. Morrison, Ralph J. DeBerardinis

**Affiliations:** ^1^ Children’s Medical Center Research Institute, University of Texas Southwestern Medical Center, Dallas, TX 75390, USA.; ^2^ Department of Molecular Biology, University of Texas Southwestern Medical Center, Dallas, TX 75390, USA.; ^3^ Howard Hughes Medical Institute, University of Texas Southwestern Medical Center, Dallas, TX 75390, USA.

## Abstract

In mice and humans with cancer, intravenous ^13^C-glucose infusion results in ^13^C labeling of tumor tricarboxylic acid (TCA) cycle intermediates, indicating that pyruvate oxidation in the TCA cycle occurs in tumors. The TCA cycle is usually coupled to the electron transport chain (ETC) because NADH generated by the cycle is reoxidized to NAD^+^ by the ETC. However, ^13^C labeling does not directly report ETC activity, and other pathways can oxidize NADH, so the ETC’s role in these labeling patterns is unverified. We examined the impact of the ETC complex I inhibitor IACS-010759 on tumor ^13^C labeling. IACS-010759 suppresses TCA cycle labeling from glucose or lactate and increases labeling from glutamine. Cancer cells expressing yeast NADH dehydrogenase-1, which recycles NADH to NAD^+^ independently of complex I, display normalized labeling when complex I is inhibited, indicating that cancer cell ETC activity regulates TCA cycle metabolism and ^13^C labeling from multiple nutrients.

## Teaser

The electron transport chain in cancer cells contributes to ^13^C labeling in vivo.

## INTRODUCTION

A great deal of effort over the past century has focused on identifying pathways of selective or enhanced importance to cancer cells. Warburg’s classical observation that slices of tumor tissue display robust lactate synthesis and disproportionately low levels of oxygen consumption relative to their rates of glucose uptake led him to hypothesize that defective respiration is a characteristic and perhaps a cause of malignancy (*1*). However, the relationship between impaired respiration and glycolytic metabolism in tumors has been questioned by more recent data indicating that mitochondrial metabolism and, particularly, the electron transport chain (ETC) are required for tumor growth in preclinical models and that many tumors appear to exhibit both glycolysis and oxidation of fuels such as glucose in vivo (*2*–*4*). In addition to oxidative phosphorylation, which produces energy in the form of adenosine triphosphate (ATP), the ETC has other metabolic functions, including the maintenance of redox balance through reoxidation of reduced electron carriers and by regulating cellular reactive oxygen species levels (*5*).

Tumors consume a variety of fuels in vivo to support energy production, synthesis of macromolecules, and redox homeostasis (*6*). Stable isotope tracing is a versatile and informative method to explore nutrient uptake and metabolism in intact biological systems, including tumors. To assess tumor metabolism in patients, we and others have provided ^13^C-labeled nutrients intravenously in the intraoperative or perioperative period leading up to surgical resection of the tumor (*7*–*9*). Extracting metabolites from the tumor and adjacent nonmalignant tissue and analyzing their ^13^C labeling provide information about which pathways are active in each compartment. Infusion of ^13^C-glucose in several kinds of human cancer, including glioblastoma, non–small cell lung cancer (NSCLC), breast cancer, and extracranial pediatric solid tumors resulted in prominent labeling of tricarboxylic acid (TCA) cycle intermediates, indicating that glucose is a respiratory fuel in these tumors (*8*–*12*). Other tumors in humans or mice transfer label to TCA cycle intermediates from other circulating nutrients, including glutamine, acetate, and lactate (*13*–*17*).

But does the appearance of labeled TCA cycle intermediates in tumors indicate that these tumors have a functional ETC? Oxidative function of the TCA cycle generates reduced electron carriers in the form of NADH [reduced form of nicotinamide adenine dinucleotide (NAD^+^)] and dihydroflavin-adenine dinucleotide (FADH_2_), and recycling these cofactors to NAD^+^ and FAD by the ETC enables persistent function of the cycle. This principle implies that fuel oxidation in the TCA cycle is coupled to respiration. However, the published isotope labeling studies in cancer have not tested this directly. A trivial alternative explanation is that labeled TCA cycle intermediates are produced elsewhere in the body and imported by tumor cells. Furthermore, other oxidoreductases and shuttling systems could, in principle, recycle electron carriers and support ^13^C labeling in TCA cycle intermediates even without the aid of the ETC (*18*).

Obtaining more clarity about the connection between the ETC and ^13^C labeling patterns is of interest because ETC blockade, for example, with the complex I inhibitor IACS-010759, is being investigated as a therapeutic approach in some cancers (*19*) and because the question of whether tumors respire in vivo is at the root of Warburg’s classical work. Therefore, the primary objective of this study was to provide data to help interpret ^13^C labeling patterns from in vivo tumor metabolism studies, in particular, to assess the role of ETC blockade by IACS-010759 on ^13^C labeling of TCA cycle intermediates and other metabolic features. We also use tumor-specific expression of enzymes that oxidize NADH to NAD^+^ independently of ETC complex I (*20*–*22*) to identify metabolic effects of IACS-010759 that can be reversed when mitochondrial NAD^+^ regeneration is reestablished in cancer cells.

## RESULTS

### Variable effects of complex I inhibition on xenograft growth

To choose models to examine the impact of complex I on tumor metabolism in vivo, we assessed the effects of IACS-010759 in a panel of cell lines. It is important to emphasize that our goal in this study was not to identify tumors with exquisite sensitivity to this drug but rather to identify tumors that grow through treatment so that the metabolic effects of ETC blockade could be separated from cytotoxic effects. We first examined IACS-010759’s effects on a panel of neuroblastoma cell lines in culture. Treatment for 72 hours resulted in variable reduction in cell count but little effect on viability other than in NB-1, which was previously reported to be highly sensitive to IACS-010759 (fig. S1, A and B) (*19*). As expected for a complex I inhibitor, IACS-010759 suppressed respiration and increased extracellular acidification even in cells that maintained viability (fig. S1, C and D).

**Fig. 1.**
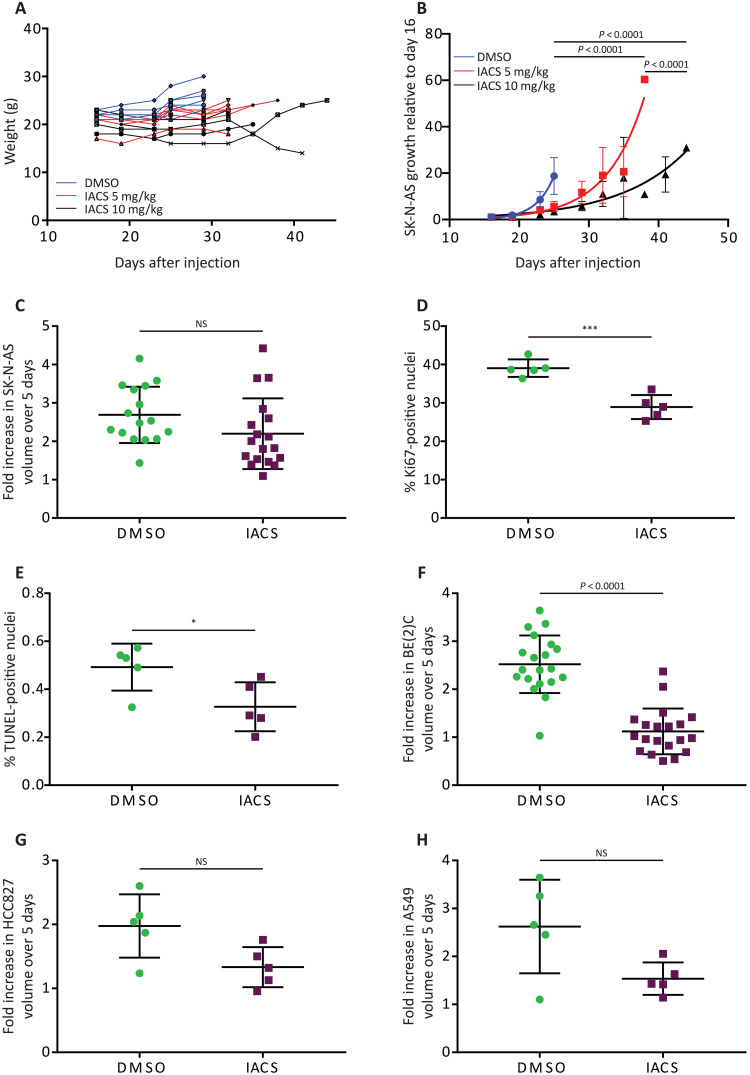
Effects of IACS-010759 on tumor growth, cell proliferation, and cell death in vivo. (**A**) Individual mouse weights over time following treatment with DMSO or IACS-010759 (5 or 10 mg/kg). *n* = 6 mice per group. (**B**) SK-N-AS tumor growth curves normalized to size at start of treatment (*n* = 6 mice per group). (**C**) Fold increase in SK-N-AS tumor volume over 5 days of treatment with DMSO or IACS-010759 (DMSO, *n* = 16; IACS-010759, *n* = 18). (**D** and **E**) Percentage of Ki67- and TUNEL-positive nuclei following treatment with DMSO or IACS-010759 (*n* = 5 mice per group, SK-N-AS xenograft). (**F** to **H**) Fold increase in tumor volume over 5 days for BE(2)C (DMSO, *n* = 20; IACS-010759, *n* = 20), HCC827 (DMSO, *n* = 5; IACS-010759, *n* = 5), and A549 (DMSO, *n* = 5; IACS-010759, *n* = 5) xenografts, treated with either DMSO or IACS-010759. Statistical significance was assessed using log_2_-transformed least squares line fitting followed by extra sum-of-square *F* tests for the differences between lines and Holm-Sidak’s method for multiple comparisons adjustment (B), log_2_-transformed two-way one-way analyses of variance (ANOVAs) followed by Sidak’s multiple comparisons adjustment (C, F, G, and H), or Student’s *t* tests followed by Holm-Sidak’s multiple comparisons adjustment (D and E). Statistical tests were two-sided. Data represent means ± SD. *P = 0.01 to 0.05 and ***P = 0.0001 to 0.001. NS, not significant.

We next generated SK-N-AS subcutaneous xenografts in non-obese diabetic (NOD)–severe combined immunodeficient *Il2rg*^−/−^ (NSG) mice and treated them with dimethyl sulfoxide (DMSO) or IACS-010759 using two previously reported regimens (*19*) until the tumors reached approximately 2 cm in diameter. Neither regimen resulted in weight loss (Fig. 1A). DMSO-treated tumors reached the size limit after 9 days of treatment, whereas tumors treated with IACS-010759 displayed more variable growth, reaching the limit after 9 to 28 days, with the higher dose (10 mg/kg) resulting in a greater response than the lower dose (5 mg/kg; Fig. 1B). Subsequent metabolomics and ^13^C infusion studies were carried out after five consecutive days of vehicle or IACS-010759 treatment (10 mg/kg), so we also measured tumor growth over this time window. SK-N-AS tumors displayed at most a mild reduction in growth over this period (Fig. 1C). The fraction of Ki67-positive nuclei was mildly reduced, but there was no increase in terminal deoxynucleotidyl transferase–mediated deoxyuridine triphosphate nick end labeling (TUNEL) staining (Fig. 1, D and E). These findings indicate that IACS-010759 at 10 mg/kg does not induce tumor cell death over this 5-day period and that the proliferating cell content is largely maintained. Three other xenograft models (one neuroblastoma and two NSCLCs) displayed variable effects on tumor growth after 5 days of treatment (Fig. 1, F to H).

### Complex I inhibition results in 3-hydroxyacylcarnitine accumulation in tumors

**Fig. 2.**
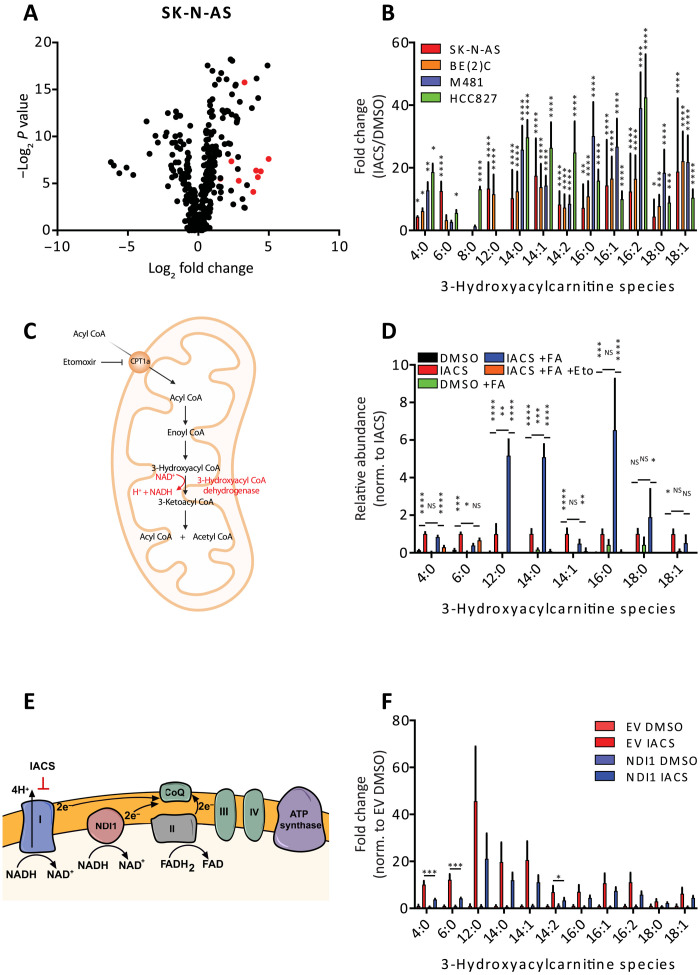
Metabolic effects of IACS-010759. (**A**) Untargeted metabolomics analysis projected as relative abundances in IACS-010759-treated versus DMSO-treated SK-N-AS xenografts (DMSO, *n* = 7; IACS-010759, *n* = 7). The 3-hydroxyacylcarnitines are indicated by red dots. (**B**) Relative abundance of 3-hydroxyacylcarnitines in four xenograft lines expressed as fold change between IACS-010759 and DMSO treatment (SK-N-AS: DMSO, *n* = 7 and IACS-010759, *n* = 7; BE(2)C: DMSO, *n* = 7 and IACS-010759, *n* = 7; M481: DMSO, *n* = 5 and IACS-010759, *n* = 5; HCC827: DMSO, *n* = 4 and IACS-010759, *n* = 4). (**C**) Schematic depicting the steps of β oxidation, including the 3-hydroxyacyl–CoA dehydrogenase–mediated conversion of 3-hydroxyacyl–CoA to 3-ketoacyl–CoA. (**D**) Relative abundance of 3-hydroxyacylcarnitines following treatment for 72 hours with 1 μM IACS-010759, 100 μM palmitate (+FA), and 5 μM etomoxir. Metabolite abundances are normalized to IACS-010759 treatment without palmitate. (**E**) Schematic showing the site of action of IACS-010759 and how NDI1 can be used to rescue complex I inhibition. (**F**) Relative abundance of 3-hydroxyacylcarnitines following NDI1 expression (EV: DMSO, *n* = 5 and IACS-010759, *n* = 6; NDI1: DMSO, *n* = 6 and IACS-010759, *n* = 5). Statistical significance was assessed using log_2_-transformed one-way ANOVA followed by Sidak’s multiple comparisons adjustment (B, D, and F), Kruskal-Wallis test followed by Dunn’s multiple comparisons adjustment (B and D), or log_2_-transformed Welch’s one-way ANOVA followed by Dunnett’s T3 method for multiple comparisons adjustment (B and F). Statistical tests were two-sided. Data represent means ± SD. *P = 0.01 to 0.05, **P = 0.001 to 0.01, ***P = 0.0001 to 0.001, and ****P < 0.0001.

To characterize the metabolic response to complex I inhibition in vivo, we first performed metabolomics on neuroblastoma [SK-N-AS and BE(2)C], melanoma (M481), and NSCLC (HCC827) xenografts in the presence and absence of IACS-010759. Primary metabolomics data for all experiments are provided in the Supplementary Materials. Metabolic differences upon IACS-010759 treatment were detected by supervised clustering (variable importance in the projection) and then consolidated into pathways using metabolite set enrichment analysis (fig. S2, A to H). As expected in the setting of ETC inhibition, all tumor models displayed alterations in central carbon pathways, including metabolism of aspartate and glutamate (fig. S2). IACS-010759–treated tumors also accumulated 3-hydroxyacylcarnitine species, which are intermediates in the β oxidation pathway (fig. S2A, C, E, and G). This effect was also observed by untargeted metabolomics analysis, which revealed a marked increase in 3-hydroxyacylcarnitine species of acyl chain lengths from 4 to 18 (Fig. 2, A and B, and fig. S3, A to C). These species arise from the second of the repeating four-step cycle of β oxidation (Fig. 2C). Their further oxidation requires the NAD^+^-dependent 3-hydroxylacyl–coenzyme A (CoA) dehydrogenases, and complex I deficiency in humans results in the accumulation of these metabolites (*23*).

Acylcarnitines are readily released into the bloodstream during β oxidation in the liver, so 3-hydroxyacylcarnitines in the tumors could result from either impaired β oxidation locally or as a consequence of the import of partial β oxidation in the liver. To explore this, we incubated SK-N-AS cells in culture with IACS-010759 and observed an increase in 3-hydroxyacylcarnitine species, particularly when the medium was supplemented with excess fatty acids (Fig. 2D). This finding demonstrates that 3-hydroxyacylcarnitines accumulate in these cells when the fatty acid availability exceeds the β oxidation capacity and suggests that 3-hydroxyacylcarnitines in vivo arise at least partially from tumor cell–autonomous fatty acid oxidation. We also observed that long-chain 3-hydroxyacylcarnitines were nearly eliminated by etomoxir, a carnitine palmitoyltransferase-1A (CPT1A) inhibitor that blocks fatty acid import into the mitochondria (Fig. 2, C and D).

Quantitative measurements of NAD^+^ and NADH from the tumors revealed a trend toward a lower NAD^+^/NADH ratio after IACS-010759 treatment, but the reduction was not statistically significant (fig. S3D). This method reports the total NAD^+^/NADH ratio in the tissue rather than the mitochondria-specific redox ratio. To test whether restoring mitochondrial NADH oxidation and electron transport can reverse the metabolic effects of IACS-010759, we expressed the yeast mitochondrial NADH dehydrogenase-1 (NDI1), which oxidizes NADH to NAD^+^, passes reducing equivalents to CoQ, and rescues perturbed redox ratios arising from complex I inhibition (Fig. 2E) (*20*, *21*). To confirm the effects of NDI1, we measured the oxygen consumption rate (OCR) and extracellular acidification rate (ECAR) in vitro and observed that NDI1 reversed the effects of complex I inhibition (fig. S3, E and F). Expression of NDI1 in SK-N-AS xenografts partially reduced the abundance of some 3-hydroxyacylcarnitines, suggesting that these tumors are capable of fatty acid oxidation (Fig. 2F). Other metabolic abnormalities caused by IACS-010759 were completely rescued in vivo by NDI1, indicating that some of these changes are tumor cell autonomous (fig. S3, G to L). NDI1 had little effect on SK-N-AS xenograft growth in the presence or absence of IACS-010759 (fig. S3M).

Accumulation of 3-hydroxyacylcarnitines is toxic in certain tissues (*24*, *25*). Palmitate increased the sensitivity of SK-N-AS cells to IACS-010759 (fig. S3N). However, this effect is probably unrelated to 3-hydroxyacylcarnitine accumulation, as etomoxir was unable to rescue the cell toxicity mediated by the addition of IACS-010759 and palmitate (fig. S3O).

### TCA cycle intermediate labeling is suppressed in vivo by IACS-010759

**Fig. 3.**
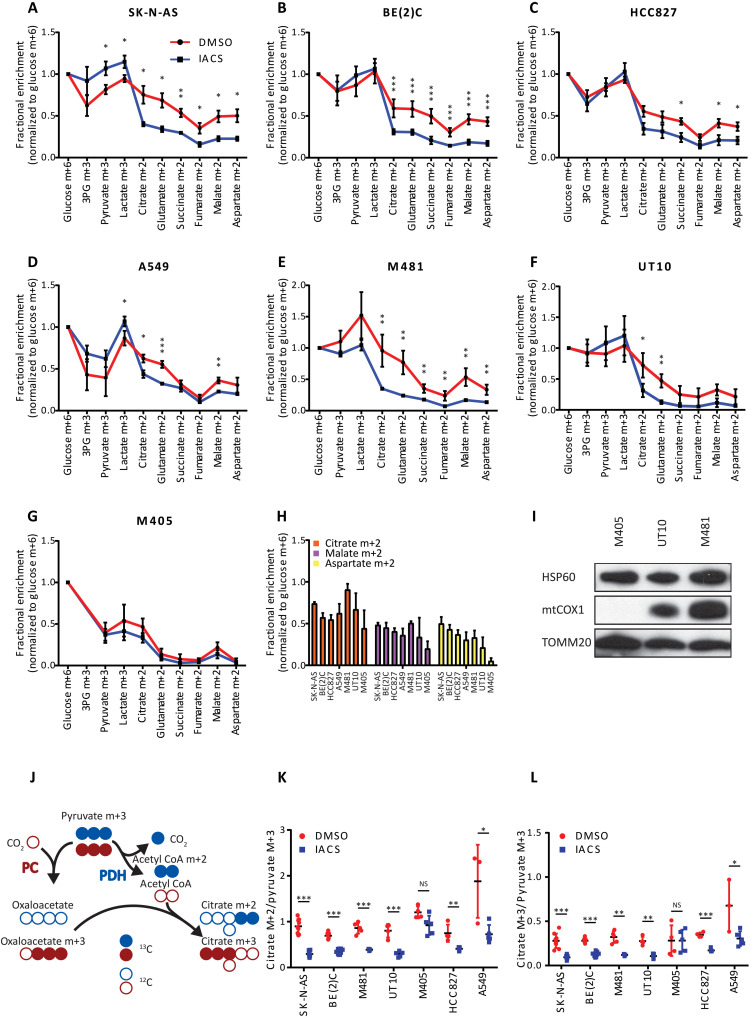
IACS-010759 reduces labeling of TCA cycle intermediates from [U-^13^C]glucose in vivo. (**A** to **G**) Fractional enrichment (normalized to glucose m+6 in tumor) in glycolytic and TCA cycle intermediates following infusion with [U-^13^C]glucose in two neuroblastoma xenograft models [SK-N-AS (A): DMSO, *n* = 3 and IACS-010759 *n* = 3; BE(2)C (B): DMSO, *n* = 5 and IACS-010759, *n* = 6], two NSCLC xenograft models [HCC827 (C): DMSO, *n* = 3 and IACS-010759, *n* = 5; A549 (D): DMSO, *n* = 3 and IACS-010759, *n* = 5], and three melanoma PDX models [M481 (E): DMSO, *n* = 5 and IACS-010759, *n* = 3; UT10 (F): DMSO, *n* = 4 and IACS-010759, *n* = 5; M405 (G): DMSO, *n* = 4 and IACS-010759, *n* = 5]. (**H**) Fractional enrichment in citrate m+2, malate m+2, and aspartate m+2 normalized to glucose m+6 across all DMSO-treated tumors. (**I**) Western blot showing absence of mtCOX1 in the melanoma M405 PDX model. (**J**) Schematic showing how ^13^C-labeled pyruvate carbons enter the TCA cycle via PDH (blue carbons) and PC (red carbons). (**K** and **L**) Effect of IACS-010759 on citrate m+2/pyruvate m+3 and citrate m+3/pyruvate m+3 ratios in all xenograft models. Statistical significance was assessed using log_2_-transformed Student’s *t* tests followed by Holm-Sidak’s multiple comparisons adjustment (A to G, K, and L). Statistical tests were two-sided. Data represent means ± SD. *P = 0.01 to 0.05, **P = 0.001 to 0.01, and ***P = 0.0001 to 0.001.

To investigate the impact of complex I inhibition on ^13^C labeling patterns in vivo, mice bearing SK-N-AS and BE(2)C neuroblastoma subcutaneous xenografts were infused with [U-^13^C]glucose after 5 days of treatment with IACS-010759. When normalized to glucose m+6, labeling in glycolytic intermediates was either maintained or enhanced in tumors pretreated with IACS-010759, but labeling in TCA cycle intermediates was suppressed (Fig. 3, A and B). We performed similar experiments in two NSCLC subcutaneous xenografts (HCC827 and A549) and three melanoma patient–derived xenografts (PDXs; M481, UT10, and M405). Again, glycolytic intermediate labeling was maintained or enhanced in the IACS-010759–treated tumors, and TCA cycle intermediate labeling was suppressed in four of the five models (Fig. 3, C to G). The lone exception was the melanoma PDX M405, in which the drug had no impact on labeling. We noted that this tumor also had relatively low labeling in TCA cycle intermediates compared to the other models, even in the untreated state (Fig. 3H). M405 cells have a single nucleotide deletion in the mitochondrial DNA–encoded gene *MT-CO1*, which encodes cytochrome C oxidase subunit 1 (COXI) of ETC complex IV (fig. S4A). The deletion results in a frameshift mutation, and Western blot analysis revealed an absence of COXI in M405 tumors (Fig. 3I). This finding indicates a genetically defined ETC abnormality in M405, likely explaining the low basal labeling of TCA cycle intermediates and lack of response to IACS-010759.

We also examined labeling in citrate and pyruvate to infer routes of ^13^C entry into the TCA cycle through pyruvate dehydrogenase (PDH) and pyruvate carboxylase (PC) (Fig. 3J). The citrate m+2/pyruvate m+3 ratio is used to indicate PDH-dependent labeling, while citrate m+3/pyruvate m+3 is used to indicate PC-dependent labeling. Both ratios declined upon treatment with IACS-010759 in every xenograft model examined except for M405 tumors (Fig. 3, K and L). Activity of the PDH complex can be regulated by inhibitory phosphorylation of the PDHA1 subunit at Ser^293^. Western blot analysis did not detect enhanced phosphorylation of this residue upon treatment with IACS-010759 (fig. S4B), consistent with PDH suppression occurring through a different mechanism.

**Fig. 4.**
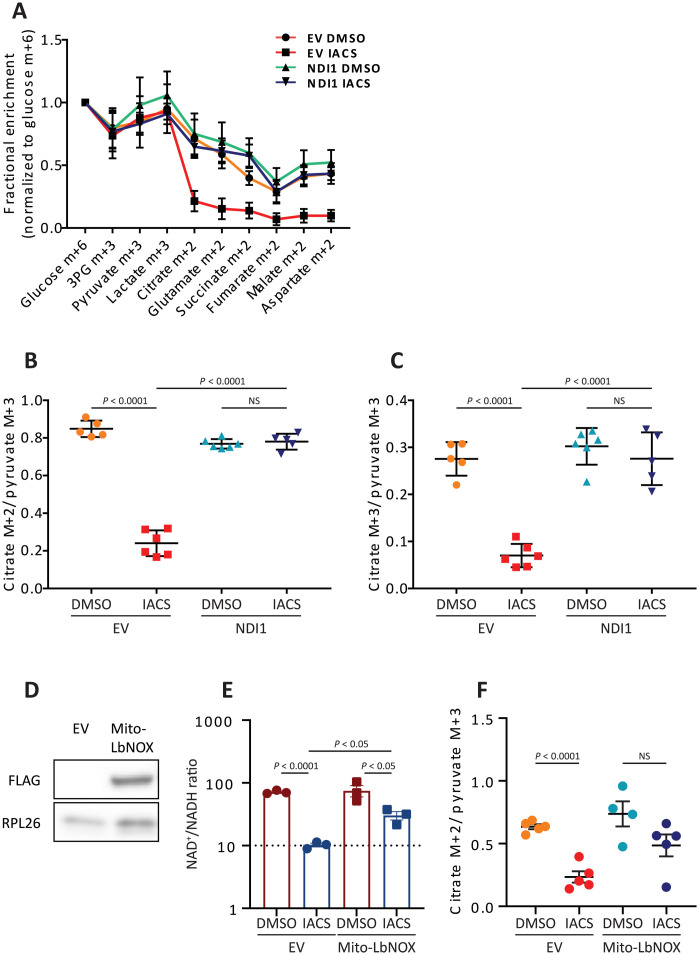
NDI1 normalizes labeling in TCA cycle intermediates following IACS-010759 treatment. (**A**) Fractional enrichment normalized to tumor glucose m+6 in SK-N-AS subcutaneous xenografts expressing NDI1 or a control vector (EV) and treated with DMSO or IACS-010759 (EV: DMSO, *n* = 5 and IACS-010759, *n* = 6; NDI1: DMSO, *n* = 6 and IACS-010759, *n* = 5). (**B**) Citrate m+2/pyruvate m+3 ratio in xenografts expressing EV or NDI1 and treated with DMSO or IACS-010759. (**C**) Citrate m+3/pyruvate m+3 ratio in xenografts expressing EV or NDI1 and treated with DMSO or IACS-010759. (**D**) Western blot showing expression of Mito-LbNOX in SK-N-AS cells. (**E**) NAD^+^/NADH ratio in cultured SK-N-AS cells expressing either EV or Mito-LbNOX and treated with DMSO or IACS-010759. (**F**) Citrate m+2/pyruvate m+3 ratios in xenografts expressing EV or Mito-LbNOX and treated with DMSO or IACS-010759. Statistical significance was assessed using one-way ANOVA followed by Sidak’s multiple comparisons adjustment (A to C) or by unpaired Student’s *t* test (D and E). Data represent means ± SD (A to C) or SEM (E and F).

Several dehydrogenases related to the TCA cycle, including PDH, isocitrate dehydrogenase, α-ketoglutarate dehydrogenase, and malate dehydrogenase require NAD^+^. If IACS-010759 suppresses TCA cycle labeling by reducing NADH recycling to NAD^+^ in cancer cells, then expressing NDI1 within cancer cells should be sufficient to increase labeling to levels in untreated tumors. We therefore repeated the [U-^13^C]glucose infusions in SK-N-AS xenografts expressing NDI1 or an empty vector (EV) control. When tumors were approximately 200 mm^3^ in volume, the mice were treated with either IACS-010759 or DMSO for 5 days and infused with [U-^13^C]glucose. In DMSO-treated mice, ^13^C labeling was essentially the same between EV and NDI1-expressing tumors (Fig. 4A). As in the parental SK-N-AS tumors, treatment with IACS-010759 suppressed fractional enrichment of TCA cycle intermediates in EV xenografts (Fig. 4A). In contrast, IACS-010759 had essentially no effect on TCA cycle intermediate labeling in NDI1-expressing tumors (Fig. 4A). Labeling ratios reflecting PDH and PC activities were also normalized by NDI1 in IACS-010759–treated tumors (Fig. 4, B and C, and fig. S4, C and D).

We next expressed a mitochondrially localized NADH oxidase from *Lactobacillus brevis* (mito-*Lb*NOX) in SK-N-AS cells (*22*). This enzyme converts NADH to NAD^+^ and transfers the reducing equivalents to O_2_, producing water. Therefore, mito-*Lb*NOX rescues NADH recycling but not mitochondrial complex I–dependent electron transport. Mito-*Lb*NOX partially rescued the NAD^+^/NADH ratio in IACS-010759–treated cells in culture (Fig. 4, D and E), and in vivo, it restored the citrate m+2/pyruvate m+3 ratio in IACS-010759–treated tumors (Fig. 4F). These observations indicate that ETC activity within tumor cells is responsible for ^13^C transfer to TCA cycle intermediates and are consistent with a model in which IACS-010759’s effects on this labeling involves a reduced NAD^+^/NADH ratio in the mitochondria.

### Complex I inhibition alters the metabolism of multiple nutrients by tumors

**Fig. 5.**
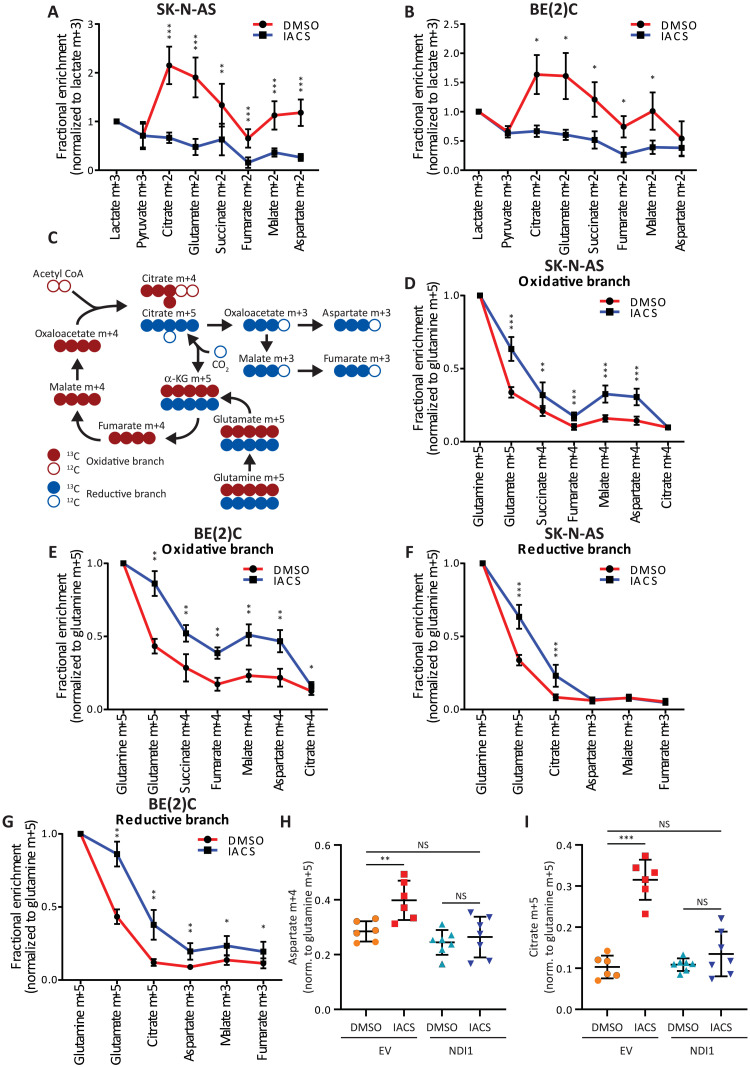
Fractional enrichment from glutamine is increased following IACS-010759 treatment. (**A** and **B**) Fractional enrichment (normalized to tumor lactate m+3) in glycolytic and TCA cycle intermediates following infusion with [U-^13^C]lactate in two neuroblastoma xenograft models [SK-N-AS (A): DMSO, *n* = 8 and IACS-010759, *n* = 9; BE(2)C (B): DMSO, *n* = 6 and IACS-010759 *n* = 6]. (**C**) Schematic showing how ^13^C-labeled glutamine carbons are metabolized either through the oxidative (red carbons) or reductive (blue carbons) pathways of the TCA cycle. (**D** and **E**) Fractional enrichment (normalized to tumor glutamine m+5) in the oxidative branch of the TCA cycle following infusion with [U-^13^C]glutamine in two neuroblastoma xenograft models [SK-N-AS (A): DMSO, *n* = 9 and IACS-010759, *n* = 9; BE(2)C (B): DMSO, *n* = 5 and IACS-010759, *n* = 8]. (**F** and **G**) Fractional enrichment (normalized to tumor glutamine m+5) in the reductive branch of the TCA cycle following infusion with [U-^13^C]glutamine in two neuroblastoma xenograft models [SK-N-AS (A): DMSO, *n* = 9 and IACS-010759, *n* = 9; BE(2)C (B): DMSO, *n* = 5 and IACS-010759, *n* = 8]. (**H** and **I**) Fractional enrichment of aspartate m+4 (normalized to tumor glutamine m+5) and citrate m+5 (normalized to tumor glutamine m+5) as read-outs of the oxidative (H) and reductive (I) TCA cycle pathways, in SK-N-AS xenograft tumors overexpressing either NDI1 or a control vector (EV). Tumors were treated with either IACS-010759 or DMSO (EV: DMSO, *n* = 6 and IACS-010759, *n* = 6; NDI1: DMSO, *n* = 7 and IACS-010759, *n* = 7). Statistical significance was assessed using Mann-Whitney tests followed by Holm-Sidak’s multiple comparisons adjustment (A, B, and D to G) or one-way ANOVA followed by Sidak’s multiple comparisons adjustment (H and I). Statistical tests were two-sided. Data represent means ± SD. *P = 0.01 to 0.05, **P = 0.001 to 0.01, and ***P = 0.0001 to 0.001.

We next explored the effect of IACS-010759 on metabolism of other nutrients. We first examined [U-^13^C]lactate because circulating lactate readily transmits label into TCA cycle intermediates in tumors and other organs (*13*, *14*). For lactate to be oxidized in the TCA cycle, it must be converted to pyruvate via lactate dehydrogenase in an NAD^+^-dependent reaction before entering the TCA cycle via either PDH or PC. This predicts that IACS-010759 would have similar effects on labeling from [U-^13^C]lactate and [U-^13^C]glucose. In both SK-N-AS and BE(2)C neuroblastoma xenografts, IACS-010759 reduced the fractional enrichment of TCA cycle intermediates (Fig. 5, A and B).

Glutamine supplies the TCA cycle in many cells (*26*–*28*). Specific routes of glutamine metabolism are influenced by activity of the TCA cycle and ETC, substrate abundance, redox homeostasis, and oxygen availability (Fig. 5C) (*28*–*33*). In particular, glutamine oxidation is a prominent feature of most cultured cancer cells when the ETC is intact, while ETC blockade increases the appearance of TCA cycle intermediates from reductive carboxylation of α-ketoglutarate derived from glutamine (*30*, *34*). We infused [U-^13^C]glutamine in mice bearing SK-N-AS or BE(2)C xenografts treated with either IACS-010759 or DMSO. All tumors under both conditions displayed labeling in glutamate and TCA cycle intermediates (Fig. 5, D and E). Labeling through the oxidative and reductive pathways can be distinguished by the number of ^13^C nuclei in the TCA cycle intermediates, with the oxidative pathway resulting in m+4 labeling and the reductive pathway producing m+5 citrate and m+3 in oxaloacetate (OAA)/aspartate, malate, and fumarate (Fig. 5C) (*30*). IACS-010759 increased labeling along the oxidative pathway in both tumors, although citrate m+4 was no different between treated and untreated mice (Fig. 5, D and E). In the reductive pathway, citrate m+5 was enhanced by IACS-010759 in both tumors, and BE(2)C tumors also displayed increased m+3 labeling in aspartate, malate, and fumarate (Fig. 5, F and G). We also performed ^13^C glutamine infusions in SK-N-AS xenografts expressing NDI1. NDI1-expressing tumors were resistant to IACS-010759–mediated changes in labeling from glutamine, with both oxidative and reductive labeling reverting to levels in DMSO-treated tumors (Fig. 5, H and I, and fig. S4, E and F). Together, these experiments demonstrate that complex I inhibition in cancer cells enhances glutamine’s contribution to the TCA cycle through multiple pathways.

## DISCUSSION

The importance of mitochondrial metabolism in tumors is becoming increasingly apparent. The major finding from the current work is that inhibiting ETC complex I suppresses ^13^C transfer from glucose to TCA cycle intermediates in several different xenograft models. Introducing NDI1 or mito-*Lb*NOX, complex I–independent enzymes that convert NADH to NAD^+^ in the mitochondria, into cancer cells reestablished TCA cycle intermediate labeling in the tumors. These are the expected results if the TCA cycle is linked to NADH recycling by complex I and other aspects of ETC function, as it is in most tissues. The significance of these findings is that they help us interpret the results of ^13^C labeling patterns in tumor metabolites from patients with cancer subjected to ^13^C infusions analogous to the ones performed here in mice. In NSCLC, gliomas, brain metastases, breast cancer, and extracranial solid tumors in children, infusion with ^13^C-glucose, ^13^C-lactate, and ^13^C-acetate results in TCA cycle labeling (*8*–*13*, *15*). Our data indicate that such labeling patterns can involve tumor cell–autonomous oxidative metabolism in the mitochondria and can be supported by the ETC.

Metabolomics analysis on IACS-010759–treated tumors was carried out across three different tumor types (neuroblastoma, NSCLC, and melanoma) with the aim of identifying generalizable metabolic changes in response to complex I inhibition. We observed across all tumors changes in central carbon pathways including glutamate and aspartate metabolism, which are intimately connected to ETC function(*35*, *36*). We also observed a notable increase in 3-hydroxyacylcarnitine species of varying chain length across all tumors. These species are the substrate of the third step of β oxidation, catalyzed by the NAD^+^-dependent 3-hydroxyacyl CoA dehydrogenases. This is the only reaction in the repeating four-step β oxidation pathway that requires NAD^+^, explaining why 3-hydroxyacylcarnitines are so sensitive to IACS-010759 treatment. Notably, related species also accumulate in patients with complex I defects (*37*). We found that cancer cells produce these intermediates in culture when confronted with IACS-010759, an effect that is enhanced with the addition of excess fatty acids. Accumulation of these species is a hallmark of certain inherited fatty acid oxidation disorders such as long-chain 3-hydroxyacyl-CoA dehydrogenase (LCHAD) deficiency, and their accumulation is speculated to be toxic in some tissues (*24*, *25*). Blocking CPT1A, which initiates mitochondrial fatty acid import, reduces 3-hydroxyacylcarnitine abundance in the presence of IACS-010759. However, CPT1A blockade did not reverse growth suppression in cells treated with palmitate and IACS-010759, indicating that other mechanisms must explain why this combination is toxic to cancer cells. Diversion of palmitate into pathways producing ceramide or other growth-suppressive lipids may be responsible for this effect.

Despite NDI1’s ability to eliminate the effects of IACS-010759 on some metabolites, it only partially reversed 3-hydroxyacylcarnintine accumulation, with the most prominent effects occurring in short-chain species. Some fatty acid oxidation enzymes physically interact with ETC supercomplexes, and in particular, the trifunctional protein that contains LCHAD activity interacts with the NADH-binding domain of complex I (*38*). This structural arrangement promotes substrate channeling and catalytic efficiency. Therefore, a possible explanation for the incomplete suppression of long-chain 3-hydroxyacylcarnitines by NDI1 is that while NDI1 enhances the conversion of NADH to NAD^+^, it lacks the physical relationship with the ETC that supports channeling of NAD^+^ to fatty acid oxidation enzymes.

Glutamine’s increased contribution to the TCA cycle is partially explained by the suppressed flow of carbon from glucose through PDH. It is interesting that glutamine oxidation persists in IACS-010759–treated tumors, as prior analyses reported increased fractional enrichment in the reductive branch but suppressed fractional enrichment in the oxidative branch following ETC impairment (*30*, *35*). Our data from IACS-010759–treated tumors are similar to persistent glutamine oxidation observed under hypoxia (*28*). It is possible that PDH and α-ketoglutarate dehydrogenase respond differently to the level of ETC dysfunction induced by IACS-010759. A lack of increased citrate m+4 during infusion with [U-^13^C]glutamine suggests incomplete turnover of the oxidative TCA cycle. This could be a consequence of decreased acetyl-CoA availability as a result of PDH inhibition, impairing the conversion of labeled OAA to citrate. OAA availability may also be reduced because acetyl-CoA activates PC (*39*). Together, these factors are predicted to increase the appearance of reductively produced citrate, which we observe as citrate m+5.

We did not set out to study durable therapeutic responses to IACS-010759 but, instead, wanted to observe the metabolic effects of this drug in the absence of major cytotoxic effects. Most models, despite prominent metabolic responses to complex I inhibition, continued to grow. This is consistent with previous work indicating that additional metabolic abnormalities (e.g., in glycolysis and related pathways) enhance dependence on oxidative phosphorylation (OxPhos) in vivo (*40*). Tumor genotype can also dictate dependence on OxPhos. It has been shown that high-risk, *MYCN*-amplified neuroblastomas are more susceptible to OxPhos inhibition (*41*, *42*), which fits with the increased growth suppression in BE(2)C (*MYCN*-amplified) tumors compared to the SK-N-AS (*MYCN* nonamplified) tumors in this study. It seems likely that if an adequate therapeutic index can be achieved for OxPhos inhibitors in cancer, efficacy will depend on metabolic flexibility of the tumors and may be restricted to distinct molecular subsets.

A limitation of the infusion approaches described so far in human cancer studies and the mouse experiments used here is that they do not quantify metabolic rates. Recent work in mouse models of cancer demonstrates that absolute TCA cycle flux may be suppressed relative to nonmalignant tissue even when labeling is rapidly transferred from circulating nutrients to TCA cycle metabolites in the tumor (*43*). Nevertheless, the current work provides evidence that tumor cells from diverse xenograft models contain ETC activity in vivo and that this activity supports labeling of TCA cycle intermediates. The reduced growth rate in the SK-N-AS model following ETC inhibition, in accordance with other in vivo studies showing reduced growth in the context of a dysfunctional ETC (*2*, *3*), demonstrates that the ETC supports maximal tumor growth in many preclinical models of cancer. We believe that documenting the relationship between the ETC and TCA cycle intermediate labeling will help recognize different patterns of ^13^C labeling observed in human tumors, some of which may have relative suppression of the ETC or components of the TCA cycle.

## MATERIALS AND METHODS

### Experimental design

To study how the ETC regulates ^13^C labeling patterns in tumors in vivo, we treated mice bearing xenografts (either cell line–derived subcutaneous xenografts or PDXs) with IACS-010759 (an ETC complex I inhibitor) and then examined metabolite abundance and isotope labeling of central carbon metabolites after infusion with ^13^C-labeled nutrients. NDI1 and mito-*Lb*Nox were expressed in cancer cells to pinpoint the role of impaired tumor cell NADH recycling on metabolic alterations brought about by IACS-010759. We focused on tumor models with the ability to grow despite IACS-010759 treatment so that we could separate the effects of ETC blockade from nonspecific metabolic effects of suppressed cancer cell growth or increased cancer cell death.

### Cell lines

Cell lines were identified using DNA fingerprinting and confirmed to be mycoplasma free using the e-Myco kit (Boca Scientific). SK-N-AS (CRL-2137) and BE(2)C (CRL-2268) cells were purchased from the American Type Culture Collection. HCC827 and A549 cells were provided by J. D. Minna, University of Texas Southwestern. SK-N-AS is a neuroblastoma cell line from a female donor with the following genetic lesions: *MYCN* nonamplified, 1p36 deletion (p36.22-36.32), 3p26 deletion (p14.2-pter), 11q23 deletion (q13.4-qter), 17q21-qter unbalanced gain (q21.31-qter), and p53 mutation (H168R) (*44*). BE(2)C is a neuroblastoma cell line from a male donor with the following genetic lesions: *MYCN* amplified, 1p36 deletion (p21.3-pter), 3p26 deletion (p14.2-pter), 11q23 aneuploidies (LOH q11-qter and gain q13.4-qter), 17q21-qter unbalanced gain (q12-qter), and p53 mutation (C135F) (*44*). HCC827 is an NSCLC (adenocarcinoma) cell line from a female donor with a mutation in *EGFR* (E746-A750 deletion). A549 is an NSCLC cell line from a male donor harboring *KRAS* and *STK11* mutations. All cells were maintained in RPMI 1640 supplemented with penicillin-streptomycin, l-glutamine (4 mM), Hepes (25 mM), and 10% fetal bovine serum at 37°C in a humidified atmosphere containing 5% CO_2_ and 95% air.

We used PMXS-NDI1 vector, a gift from D. Sabatini (Addgene, plasmid no. 72876) (*45*), to generate the stable NDI1-expressing SK-N-AS cell line. PMXS-NDI1 or PMXS EVs with packaging vectors Gag-Pol and vesicular stomatitis virus glycoprotein (VSVG) were transfected into 293T cells with the ratio 4:3:1 (PMXS/Gag-Pol/VSVG) using Lipofectamine 2000 (Invitrogen, 11668027). We used PMXS-MitoLbNOX vector, a gift from J. Garcia-Bermudez (*46*), to generate the stable MitoLbNOX-expressing SK-N-AS cell line. PMXS-MitoLbNOX or PMXS EVs with packaging vectors Gag-Pol and VSVG were transfected into 293T cells with the ratio 2:2:1 (PMXS/Gag-Pol/VSVG) using Lipofectamine 3000 (Thermo Fisher Scientific, L3000015). Media containing the viruses were collected and filtered using 0.45-μm membrane 48 hours after transfection. SK-N-AS cells were cultured in the media containing lentivirus and polybrene (4 μg/ml; Sigma-Aldrich, TR-1003-G) for 24 hours followed by blasticidin (10 μg/ml) selection until all the uninfected control cells died.

### Proliferation and cell viability assays

Cell proliferation and viability were measured using a Celigo Image Cytometer. Briefly, cells were plated in 96-well plates and stained with propidium iodide and Hoechst 33342 (both used at a final concentration of 1 mg/ml) for 30 min. Hoechst 33342–positive cells were quantified for the total cell count, and cell viability was calculated as the fraction of propidium iodide–negative cells divided by the total number of cells. Cell viability was also assayed using flow cytometry. Cells were washed with staining medium and resuspended in 4′,6-diamidino-2-phenylindole (1 μg ml^−1^; Sigma-Aldrich) to differentiate live from dead cells. Cells were analyzed using an LSRFortessa cell analyzer (Becton Dickinson).

### Immunoblotting

Tumors were dissociated with disposable pestles using radioimmunoprecipitation assay buffer (Cell Signaling Technology) supplemented with protease and phosphatase inhibitor cocktails (Roche). The bicinchoninic acid protein assay (Thermo Fisher Scientific) was used to quantify protein concentration. Equal amounts of protein (10 to 20 μg) were loaded into each lane and separated on 4 to 20% polyacrylamide tris glycine SDS gels (Bio-Rad) and then transferred to polyvinylidene difluoride membranes (Bio-Rad). The membranes were blocked for 1 hour at room temperature with 5% milk in tris-buffered saline supplemented with 0.1% Tween-20 and then incubated with primary antibodies overnight at 4°C. After washing and then incubating with horseradish peroxidase–conjugated secondary antibody (Cell Signaling Technology), signals were developed using Pierce ECL or SuperSignal West (Thermo Fisher Scientific). Blots were sometimes stripped using Restore stripping buffer (Thermo Fisher Scientific) and reprobed with other primary antibodies. The following antibodies were used for Western blots: anti-PDH (C54G1, Cell Signaling Technology), anti–phospho-PDHα1 (Ser^293^; 31866, Cell Signaling Technology), anti–cyclophilin B (D1V5J; 43603, Cell Signaling Technology), anti-S6K (92025, Cell Signaling Technology), anti-calnexin (AD2-SPA-860-F, Enzo), anti-HSP60 (15282-1-AP, Proteintech), anti-MTCO1 (1D6E1A8, Abcam), anti-TOM20 (11802-1-AP, Proteintech), anti-Flag (F3165, Sigma-Aldrich), and anti-RPL26 (A305-010A-T, Bethyl Laboratories).

### Histology

Tumors were dissected, fixed in 10% formalin, sectioned, and stained for Ki67- and TUNEL-positive nuclei. Slides were imaged using an inverted Zeiss LSM 780 confocal. Ki67- and TUNEL-positive nuclei were quantified using ImageJ.

### Seahorse XFe96 respirometry

OCR and ECAR were measured using an XFe96 extracellular flux (XF) analyzer (Seahorse Bioscience). Briefly, cells were plated at 2 × 10^4^ per well in 80 μl of RPMI 1640 medium with 4 mM glutamine. Cells were incubated in a CO_2_-free incubator at 37°C for 1 hour to allow for temperature and pH equilibration before loading into the XFe96 instrument. XF assays consisted of three mix (3 min) and measurement (3 min) cycles, allowing for determination of OCR/ECAR every 6 min.

### Mouse and xenograft assays

All mouse experiments complied with relevant ethical regulations and were performed according to protocols approved by the Institutional Animal Care and Use Committee at the University of Texas Southwestern Medical Center (protocol 2016-101360 and 2016-101694). To establish xenografts from cancer cell lines, suspensions of neuroblastoma or NSCLC cells were prepared for injection in RPMI 1640 medium with Matrigel (CB-40234, Fisher Scientific). A 1:1 volume ratio was used with 50 μl of the cell suspension in RPMI 1640 medium together with 50 μl of Matrigel for a total volume of 100 μl per mouse. Subcutaneous injections were performed in the right flank of NOD.Cg-*Prkdc^scid^ Il2rg^tm1Wjl^*/SzJ (NSG) mice. Six- to eight-week-old female NSG mice were transplanted with 1 million tumor cells. Mice were randomized between treatments. For studies that involved treatment with the complex I inhibitor (IACS-010759, ChemieTek), when subcutaneous tumors reached 200 mm^3^ in volume, the mice were administered IACS-010759 by oral gavage every day for 5 days [10 mg/kg body mass in 100 μl of 0.5% methylcellulose and 4% DMSO, adapted from (*19*)]. On the fifth day, mice were infused (see below) and tumors were collected 5 hours following the last treatment dose. Mice were weighed, and subcutaneous tumors were measured at the beginning and end of the 5-day treatment period.

Melanoma specimens were obtained with informed consent from all patients according to protocols approved by the Institutional Review Board (IRB) of the University of Michigan Medical School [IRBMED approvals HUM00050754 and HUM00050085; see (*47*)] and the University of Texas Southwestern Medical Center (IRB approval 102010-051). Single-cell suspensions were obtained by dissociating tumors mechanically with a scalpel on ice. Cells were filtered through a 40-μm cell strainer to remove clumps.

For studies in which mice were treated with IACS-010759 for 5 days, tumor volume was measured at the start and the end of the 5-day treatment window. For tumor growth curves, mouse weights and tumor volume were measured every 3 days. Tumor volume was calculated by taking two orthogonal measurements of tumor diameter and then using the formula: [*L*_1_ × (*L*_2_^2^)]/2 to estimate three-dimensional tumor volume.

### Infusions with ^13^C-labeled nutrients

Mice were not fasted for these experiments. Catheters (25-gauge) were placed in the lateral tail vein under anesthesia. Isotope infusions were started immediately after implantation of the catheter and continued for approximately 3 hours, also under anesthesia. In the glucose infusions, the total dose of glucose was 2.48 g/kg and dissolved in 750 μl of saline. The glucose solution was administered as a bolus (125 μl/min over 1 min) followed by a continuous rate of 2.5 μl/min for 3 hours. The total dose of glutamine was 1.73 g/kg dissolved in 1500 μl of saline. The glutamine solution was administered as a bolus (150 μl/min over 1 min) followed by 2.5 μl/min for 5 hours. Lactate was purchased as a liquid (20% w/w), and the dose was 1.44 g/kg. This was administered as a bolus (15 μl/min over 10 min) followed by a continuous rate of 2 μl/min for 3 hours. Blood samples of ∼20 μl were obtained every 30 min via retro-orbital bleed. Animals were euthanized at the end of the infusion; then, tumors were harvested, rinsed briefly in cold saline, and frozen in liquid nitrogen.

### Gas chromatography–mass spectrometry analysis

Plasma samples were thawed on ice; then, 10 to 20 μl of each sample was added to 1 ml of 80% methanol for extraction. Frozen tumor samples of 20 to 30 mg were added to 1 ml of 80% methanol and manually homogenized. All samples were subjected to three freeze-thaw cycles followed by centrifugation at 14,000 rpm for 10 min. The supernatant was dried down using a vacuum concentrator and resuspended in 40 μl of methoxyamine (10 mg/ml) in pyridine. These samples were transferred to autoinjector vials and heated at 70°C for 15 min. A total of 70 μl of tert-butyldimethylsilyl was then added, and the samples were briefly vortexed and heated for another 60 min at 70°C. Injections of 1 μl were analyzed on an Agilent 7890A gas chromatograph coupled to an Agilent 5975C mass selective detector. The data were then corrected for natural abundance to determine mass isotopologue distributions for informative metabolites (*48*).

### Metabolomics analysis

Hydrophilic interaction liquid chromatography (HILIC) chromatographic separation of metabolites was achieved using a Millipore ZIC-pHILIC column (5 μm, 2.1 mm by 150 mm) with a binary solvent system of 10 mM ammonium acetate in water (pH 9.8) (solvent A) and acetonitrile (solvent B) with a constant flow rate of 0.25 ml min^−1^. For gradient separation, the column was equilibrated with 90% solvent B. After injection, the gradient proceeded as follows: 0- to 15-min linear ramp from 90% B to 30% B, 15- to 18-min isocratic flow of 30% B, 18- to 19-min linear ramp from 30% B to 90% B, and 19- to 27-min column regeneration with isocratic flow of 90% B. Metabolites were measured with a Thermo Scientific QExactive HF-X hybrid quadrupole orbitrap high-resolution mass spectrometer (HRMS) coupled to a Vanquish UHPLC. HRMS data were acquired with two separate acquisition methods. Individual samples were acquired with an HRMS full scan (precursor ion only) method switching between positive and negative polarities. For data-dependent, high-resolution tandem mass spectrometry (ddHRMS/MS) methods, precursor ion scans were acquired at a resolving power of 120,000 full width at half-maximum (FWHM) with a mass range of either 50 to 750 Da or 70 to 1050 Da. The Automatic gain control (AGC) target value was set to 1 × 10^6^ with a maximum injection time of 100 ms. Pooled samples were generated from an equal mixture of all individual samples and analyzed using individual positive- and negative-polarity spectrometry ddHRMS/MS acquisition methods for high-confidence metabolite ID. Product ion spectra were acquired at a resolving power of 15,000 FWHM without a fixed mass range. The AGC target value was set to 2 × 10^5^ with a maximum injection time of 150 ms. Data-dependent parameters were set to acquire the top 10 ions with a dynamic exclusion of 30 s and a mass tolerance of 5 parts per million (ppm). Isotope exclusion was turned on, and a normalized collision energy value of 30 was used, or a stepped-normalized collision energy was applied with values of 30, 50, and 70. Settings remained the same in both polarities. Metabolite identities were confirmed in three ways: (i) Precursor ion mass/charge ratio was matched within 5 ppm of theoretical mass predicted by the chemical formula. (ii) Fragment ion spectra were matched within a 5-ppm tolerance to known metabolite fragments. (iii) The retention time of metabolites was within 5% of the retention time of a purified standard run with the same chromatographic method. Metabolites were relatively quantitated by integrating the chromatographic peak area of the precursor ion searched within a 5-ppm tolerance.

### NAD^+^/NADH measurement by liquid chromatography–tandem mass spectrometry

Quantitative analysis of NAD^+^/NADH levels was performed as described previously (*16*) on a QExactive HF-X mass spectrometer (Thermo Scientific, Bremen, Germany). Quantitative analysis of NAD^+^/NADH was adapted from a previous method (*49*) and performed on a 6500+ mass spectrometer (AB Sciex, Framingham, MA). To prepare quantitative samples, tissue was lysed on ice using a plastic homogenizer in a tube containing 200 μl of 40:40:20 acetonitrile:methanol:water (v/v) with 0.1 M formic acid. Samples were vortexed for 10 s then chilled on ice for 3 min. Each tube was neutralized with 17.4 μl of 15% NH_4_HCO_3_ (w/v) in water and then quickly vortexed and placed on dry ice for 20 min. Samples were then centrifuged at 16,000*g* for 15 min at 4°C, and the supernatant was collected. ^15^N_5_ Adenosine monophosphate (AMP) internal standard was added to each extract at the final concentration of 100 nM, and the samples were analyzed on a triple quadrupole mass spectrometer (AB Sciex QTRAP 6500) on the day of extraction to prevent metabolite degradation.

Separation of analytes was achieved using another previously reported method (*50*) using a reversed-phase C18 column (Waters HSS T3, 50 mm by 2.1 mm, 1.8 μm) on an Eksigent LC20A UHPLC module. A binary gradient composed of water/methanol (95:5, v/v) with 4 mM dibutylammonium acetate (solvent A) and water/acetonitrile (25:75, v/v, solvent B). The initial solvent composition was 0% B, which was linearly ramped to 80% B over 3.2 min; from here, the gradient was increased linearly from 80% B to 100% B over 2 min. One hundred percent B was flowed isocratically for 1.2 min and then returned to 0% B for 1.5 min to reequilibrate the column. Solvent was flowed at a constant rate of 0.15 ml/min.

The mass spectrometer was operated in multiple reaction monitoring mode with polarity switching. Source gasses and mass transitions were optimized manually for all analytes with a T-infusion of a purified standard and mobile phase with a total flow rate of 0.15 μl/min. We optimized the following source conditions: The curtain gas was set to 20, and the collision gas was set to high. The ion spray voltage was set to −4500 in negative mode and 5500 in positive mode. The source temperature was set to 400. Ion source gas 1 was set to 20, while ion source gas 2 was set to 5. We found that NAD^+^ ionized best in positive ionization mode, and NADH ionized best in negative ionization mode. The ^15^N_5_-AMP internal standard was monitored in both positive and negative ionization modes. Two transitions were monitored for each analyte to confirm its identity, but only one transition was used for quantitation. For NAD^+^, 664/428 and 664/524 were monitored; 664/428 was used for quantitation. For NADH, 664/397 and 664/408 were monitored; 664/397 was used for quantitation. For ^15^N_5_-AMP, 353/141 was monitored in positive mode, while 351/139 was monitored in negative mode. For quantitation, NAD^+^ was normalized to the signal of ^15^N_5_-AMP in positive mode, while NADH quantitation was normalized to the ^15^N_5_-AMP signal in negative mode. All cellular extracts were analyzed against an eight-point standard curve ranging from 5 to 1000 nM. All standard curves had coefficient of determination values greater than or equal to 0.98 with greater than six calibrators having accuracies within 20% of their known concentration.

### Statistical methods

In each type of experiment, multiple mice were tested in multiple independent experiments performed on different days. Mice were allocated to experiments randomly, and samples were processed in an arbitrary order, but formal randomization techniques were not used. No formal blinding was applied when performing the experiments or analyzing the data. Sample sizes were not predetermined on the basis of statistical power calculations but were based on our experience with these assays. No data were excluded.

Before analyzing the statistical significance of differences among treatment groups, we tested whether the data was normally distributed and whether variance was similar among groups. To test for normality, we performed the Shapiro-Wilk tests when 3 ≤ *n* < 20 or D’Agostino Omnibus tests when *n* ≥ 20. To test whether variability significantly differed among groups, we performed *F* tests (for experiments with two groups) or Levene’s median tests (for experiments with more than two groups). When the data significantly deviated from normality or variability significantly differed among groups, we log_2_-transformed the data and tested again for normality and variability. If the transformed data no longer significantly deviated from normality and equal variability, we performed parametric tests on the transformed data. If log_2_ transformation was not possible or the transformed data still significantly deviated from normality or equal variability, we performed nonparametric tests on the nontransformed data.

When data or log_2_-transformed data were normal and equally variable, statistical analyses were performed using Student’s *t* tests (when there were two groups), one-way analyses of variance (ANOVAs; when there were more than two groups), or two-way ANOVAs (when there were two or more groups with multiple subcategories within each group). When the data or log_2_-transformed data were normal but unequally variable, statistical analyses were performed using Welch’s *t* tests (when there were two groups) or Welch’s one-way ANOVAs (when there were more than two groups). When the data and log_2_-transformed data were abnormal or unequally variable, statistical analysis was performed using Mann-Whitney tests (when there were two groups) or Kruskal-Wallis tests (when there were more than two groups). *P* values from multiple comparisons were adjusted using Sidak’s (when there were more than two groups and planned comparisons) or Dunnett’s method (when there were more than two groups and comparisons were between the control and other groups) after ANOVA, Dunnett’s T3 method after Welch’s one-way ANOVA, or Dunn’s method after Kruskal-Wallis test. For the tumor growth data over time, we used straight-line least squares fitting on the log_2_-transformed data. Holm-Sidak’s method was used to adjust comparisons involving multiple metabolites or cell lines between two treatments or among multiple tumor growth curves. All statistical tests were two-sided. All data represent means ± SD. Statistical tests were performed using GraphPad Prism V9.2.0 or R 4.0.2.

## Notes

View/request a protocol for this paper from *Bio-protocol*.
