# Supplementary material for: In vivo isotope tracing reveals a requirement for the electron transport chain in glucose and glutamine metabolism by tumors
Source: Sci Adv. 2022 Aug 31;8(35):eabn9550. doi: 10.1126/sciadv.abn9550 (PMC9432826; doi:10.1126/sciadv.abn9550)
Supplement: Supplementary file 1 — Figs. S1 to S4 [file sciadv.abn9550_sm.pdf]

Science Advances
AAAS

Supplementary Materials for

In vivo isotope tracing reveals a requirement for the electron transport chain in glucose and glutamine metabolism by tumors

Panayotis Pachnis *et al.*

Corresponding author: Ralph J. DeBerardinis, ralph.deberardinis@utsouthwestern.edu

Sci. Adv. **8**, eabn9550 (2022)
DOI: 10.1126/sciadv.abn9550

The PDF file includes:

Figs. S1 to S4

Other Supplementary Material for this manuscript includes the following:

Data files S1 and S2

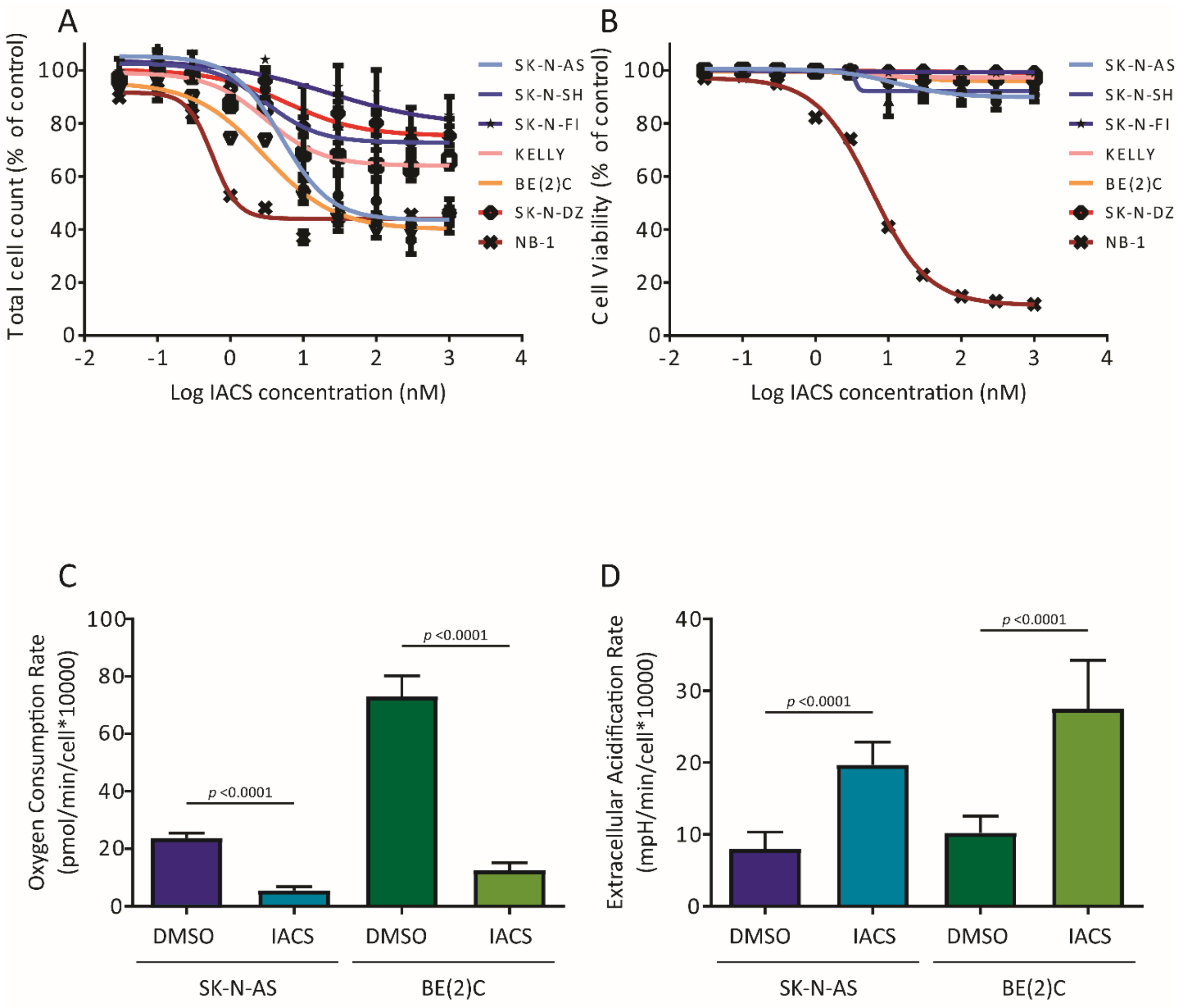
A
Total cell count (% of control)
Log IACS concentration (nM)
SK-N-AS
SK-N-SH
SK-N-FI
KELLY
BE(2)C
SK-N-DZ
NB-1
B
Cell Viability (% of control)
Log IACS concentration (nM)
C
Oxygen Consumption Rate (pmol/min/cell*10000)
p <0.0001
DMSO
IACS
SK-N-AS
BE(2)C
D
Extracellular Acidification Rate (mpH/min/cell*10000)
p <0.0001
DMSO
IACS
SK-N-AS
BE(2)C

Figure S1: **IACS-010759 suppresses OXPHOS in vitro**

(A) Total cell count as a percentage of untreated control cells, across a panel of 7 neuroblastoma cell lines. Data are expressed as average and SD.

(B) Viability as a percentage of untreated control cells, across a panel of 7 neuroblastoma cell lines. Data are expressed as average and SD.

(C and D) Oxygen consumption and extracellular acidification rates in SK-N-AS and BE(2)C cells treated with 100nM IACS-010759 or DMSO. Data are expressed as average and SD.

Statistical significance was assessed using $\log_2$-transformed one-way ANOVA followed by Sidak's multiple comparisons adjustment (C, D). Statistical tests were two-sided. Data represent mean ± SD. *p = 0.01 - 0.05, **p = 0.001 - 0.01, ***p = 0.0001 - 0.001.

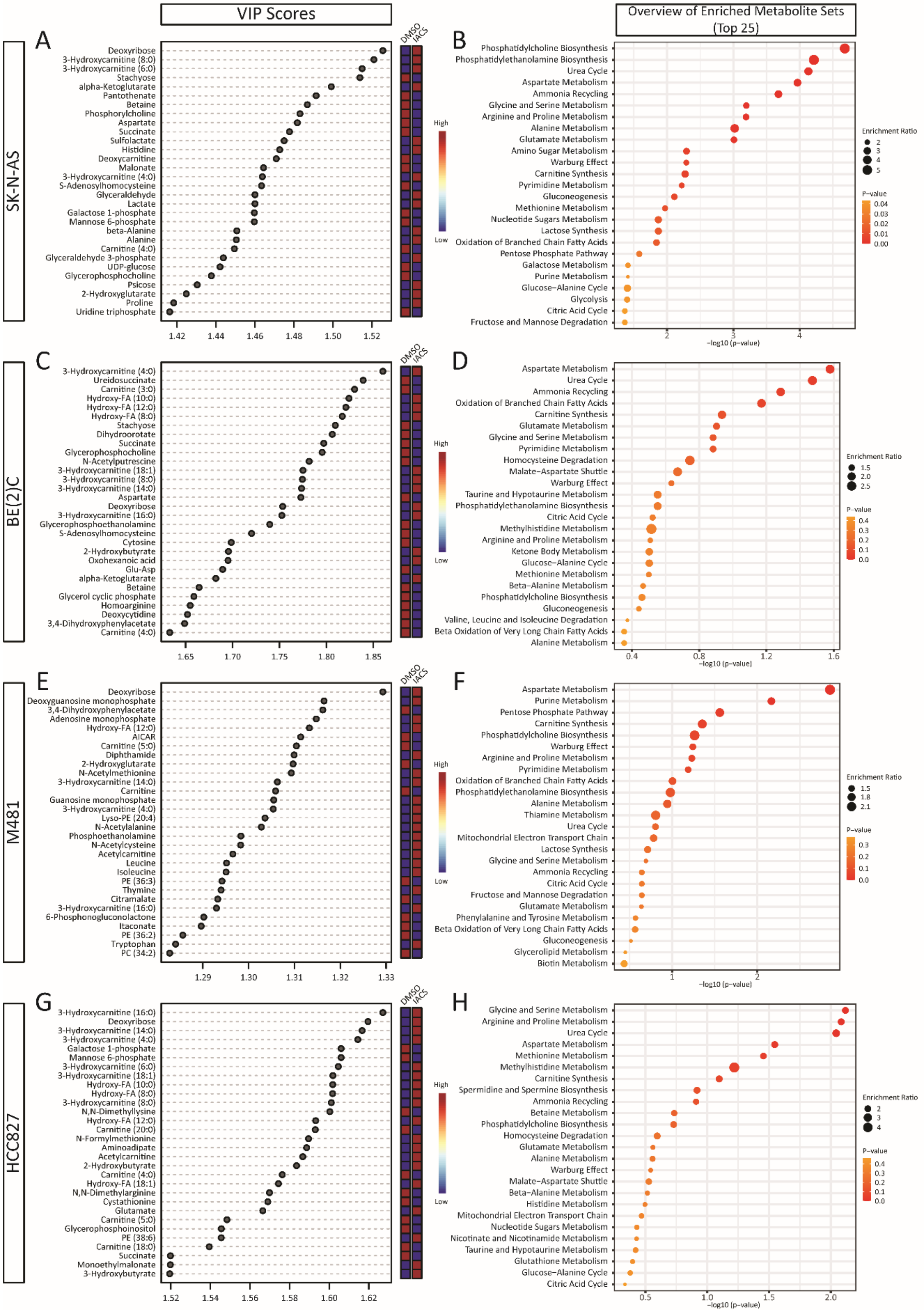
VIP Scores
Overview of Enriched Metabolite Sets (Top 25)
SK-N-AS
A
Deoxyribose
3-Hydroxycarnitine (8:0)
3-Hydroxycarnitine (6:0)
Stachyose
alpha-Ketoglutarate
Pantothenate
Betaine
Phosphorylcholine
Aspartate
Succinate
Sulfolactate
Histidine
Deoxycarnitine
Malonate
3-Hydroxycarnitine (4:0)
S-Adenosylhomocysteine
Glyceraldehyde
Lactate
Galactose 1-phosphate
Mannose 6-phosphate
beta-Alanine
Alanine
Carnitine (4:0)
Glyceraldehyde 3-phosphate
UDP-glucose
Glycerophosphocholine
Psicose
2-Hydroxyglutarate
Proline
Uridine triphosphate
1.42 1.44 1.46 1.48 1.50 1.52
DMSO IACS
High
Low
B
Phosphatidylcholine Biosynthesis
Phosphatidylethanolamine Biosynthesis
Urea Cycle
Aspartate Metabolism
Ammonia Recycling
Glycine and Serine Metabolism
Arginine and Proline Metabolism
Alanine Metabolism
Glutamate Metabolism
Amino Sugar Metabolism
Warburg Effect
Carnitine Synthesis
Pyrimidine Metabolism
Gluconeogenesis
Methionine Metabolism
Nucleotide Sugars Metabolism
Lactose Synthesis
Oxidation of Branched Chain Fatty Acids
Pentose Phosphate Pathway
Galactose Metabolism
Purine Metabolism
Glucose−Alanine Cycle
Glycolysis
Citric Acid Cycle
Fructose and Mannose Degradation
2 3 4
−log10 (p−value)
Enrichment Ratio
2 3 4 5
P−value
0.04 0.03 0.02 0.01 0.00
BE(2)C
C
3-Hydroxycarnitine (4:0)
Ureidosuccinate
Carnitine (3:0)
Hydroxy-FA (10:0)
Hydroxy-FA (12:0)
Hydroxy-FA (8:0)
Stachyose
Dihydroorotate
Succinate
Glycerophosphocholine
N-Acetylputrescine
3-Hydroxycarnitine (18:1)
3-Hydroxycarnitine (8:0)
3-Hydroxycarnitine (14:0)
Aspartate
Deoxyribose
3-Hydroxycarnitine (16:0)
Glycerophosphoethanolamine
S-Adenosylhomocysteine
Cytosine
2-Hydroxybutyrate
Oxohexanoic acid
Glu-Asp
alpha-Ketoglutarate
Betaine
Glycerol cyclic phosphate
Homoarginine
Deoxycytidine
3,4-Dihydroxyphenylacetate
Carnitine (4:0)
1.65 1.70 1.75 1.80 1.85
DMSO IACS
High
Low
D
Aspartate Metabolism
Urea Cycle
Ammonia Recycling
Oxidation of Branched Chain Fatty Acids
Carnitine Synthesis
Glutamate Metabolism
Glycine and Serine Metabolism
Pyrimidine Metabolism
Homocysteine Degradation
Malate−Aspartate Shuttle
Warburg Effect
Taurine and Hypotaurine Metabolism
Phosphatidylethanolamine Biosynthesis
Citric Acid Cycle
Methylhistidine Metabolism
Arginine and Proline Metabolism
Ketone Body Metabolism
Glucose−Alanine Cycle
Methionine Metabolism
Beta−Alanine Metabolism
Phosphatidylcholine Biosynthesis
Gluconeogenesis
Valine, Leucine and Isoleucine Degradation
Beta Oxidation of Very Long Chain Fatty Acids
Alanine Metabolism
0.4 0.8 1.2 1.6
−log10 (p−value)
Enrichment Ratio
1.5 2.0 2.5
P−value
0.4 0.3 0.2 0.1 0.0
M481
E
Deoxyribose
Deoxyguanosine monophosphate
3,4-Dihydroxyphenylacetate
Adenosine monophosphate
Hydroxy-FA (12:0)
AICAR
Carnitine (5:0)
Diphthamide
2-Hydroxyglutarate
N-Acetylmethionine
3-Hydroxycarnitine (14:0)
Carnitine
Guanosine monophosphate
3-Hydroxycarnitine (4:0)
Lyso-PE (20:4)
N-Acetylalanine
Phosphoethanolamine
N-Acetylcysteine
Acetylcarnitine
Leucine
Isoleucine
PE (36:3)
Thymine
Citramalate
3-Hydroxycarnitine (16:0)
6-Phosphogluconolactone
Itaconate
PE (36:2)
Tryptophan
PC (34:2)
1.29 1.30 1.31 1.32 1.33
DMSO IACS
High
Low
F
Aspartate Metabolism
Purine Metabolism
Pentose Phosphate Pathway
Carnitine Synthesis
Phosphatidylcholine Biosynthesis
Warburg Effect
Arginine and Proline Metabolism
Pyrimidine Metabolism
Oxidation of Branched Chain Fatty Acids
Phosphatidylethanolamine Biosynthesis
Alanine Metabolism
Thiamine Metabolism
Urea Cycle
Mitochondrial Electron Transport Chain
Lactose Synthesis
Glycine and Serine Metabolism
Ammonia Recycling
Citric Acid Cycle
Fructose and Mannose Degradation
Glutamate Metabolism
Phenylalanine and Tyrosine Metabolism
Beta Oxidation of Very Long Chain Fatty Acids
Gluconeogenesis
Glycerolipid Metabolism
Biotin Metabolism
1 2
−log10 (p−value)
Enrichment Ratio
1.5 1.8 2.1
P−value
0.3 0.2 0.1 0.0
HCC827
G
3-Hydroxycarnitine (16:0)
Deoxyribose
3-Hydroxycarnitine (14:0)
3-Hydroxycarnitine (4:0)
Galactose 1-phosphate
Mannose 6-phosphate
3-Hydroxycarnitine (6:0)
3-Hydroxycarnitine (18:1)
Hydroxy-FA (10:0)
Hydroxy-FA (8:0)
3-Hydroxycarnitine (8:0)
N,N-Dimethyllysine
Hydroxy-FA (12:0)
Carnitine (20:0)
N-Formylmethionine
Aminoadipate
Acetylcarnitine
2-Hydroxybutyrate
Carnitine (4:0)
Hydroxy-FA (18:1)
N,N-Dimethylarginine
Cystathionine
Glutamate
Carnitine (5:0)
Glycerophosphoinositol
PE (38:6)
Carnitine (18:0)
Succinate
Monoethylmalonate
3-Hydroxybutyrate
1.52 1.54 1.56 1.58 1.60 1.62
DMSO IACS
High
Low
H
Glycine and Serine Metabolism
Arginine and Proline Metabolism
Urea Cycle
Aspartate Metabolism
Methionine Metabolism
Methylhistidine Metabolism
Carnitine Synthesis
Spermidine and Spermine Biosynthesis
Ammonia Recycling
Betaine Metabolism
Phosphatidylcholine Biosynthesis
Homocysteine Degradation
Glutamate Metabolism
Alanine Metabolism
Warburg Effect
Malate−Aspartate Shuttle
Beta−Alanine Metabolism
Histidine Metabolism
Mitochondrial Electron Transport Chain
Nucleotide Sugars Metabolism
Nicotinate and Nicotinamide Metabolism
Taurine and Hypotaurine Metabolism
Glutathione Metabolism
Glucose−Alanine Cycle
Citric Acid Cycle
0.5 1.0 1.5 2.0
−log10 (p−value)
Enrichment Ratio
2 3 4
P−value
0.4 0.3 0.2 0.1 0.0

Figure S2: Metabolic changes following treatment with IACS-010759

(A, C, E and G) Partial Least Squares Discriminant Analysis (PLS-DA) showing the 30 metabolites with the highest Variable Importance in Projection (VIP) score for each tumor type, comparing DMSO and IACS-010759-treated tumors (SK-N-AS DMSO n=7, IACS-010759 n=7; BE(2)C DMSO n=7, IACS-010759 n=7; M481 DMSO n=5, IACS-010759 n=5; HCC827 DMSO n=4, IACS-010759 n=4).

(B, D, F and H) Metabolite Set Enrichment Analysis (MSEA) performed on all metabolites with VIP scores >1 for each tumor type, comparing DMSO and IACS-010759-treated tumors (SK-N-AS DMSO n=7, IACS-010759 n=7; BE(2)C DMSO n=7, IACS-010759 n=7; M481 DMSO n=5, IACS-010759 n=5; HCC827 DMSO n=4, IACS-010759 n=4).

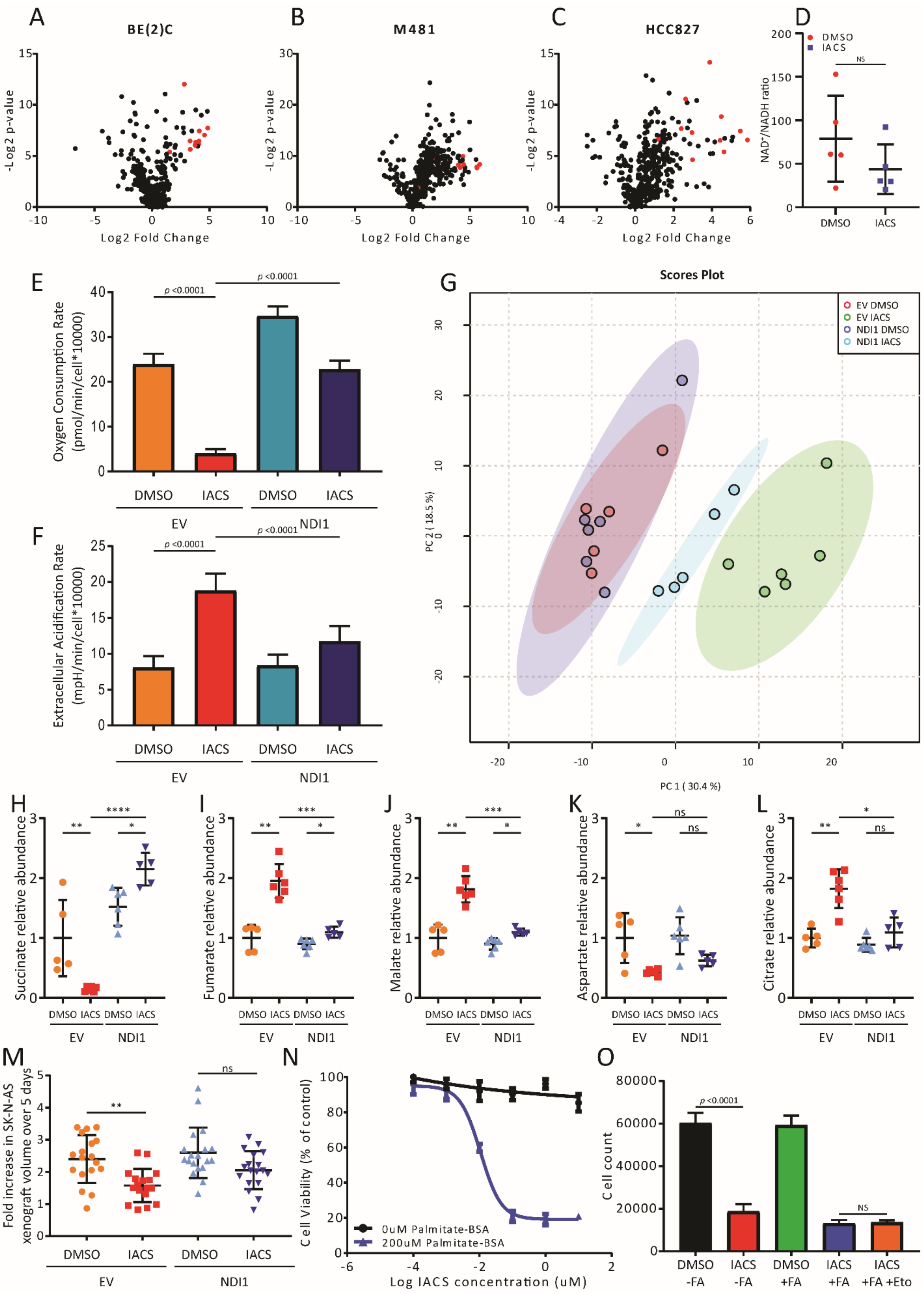
A
BE(2)C
-Log2 p-value
Log2 Fold Change
B
M481
-Log2 p-value
Log2 Fold Change
C
HCC827
-Log2 p-value
Log2 Fold Change
D
DMSO
IACS
NS
NAD+/NADH ratio
DMSO
IACS
E
Oxygen Consumption Rate (pmol/min/cell*10000)
p <0.0001
p <0.0001
DMSO
IACS
DMSO
IACS
EV
NDI1
F
Extracellular Acidification Rate (mpH/min/cell*10000)
p <0.0001
p <0.0001
DMSO
IACS
DMSO
IACS
EV
NDI1
G
Scores Plot
EV DMSO
EV IACS
NDI1 DMSO
NDI1 IACS
PC 2 (18.5 %)
PC 1 (30.4 %)
H
Succinate relative abundance
**
****
*
DMSO IACS DMSO IACS
EV
NDI1
I
Fumarate relative abundance
**
***
*
DMSO IACS DMSO IACS
EV
NDI1
J
Malate relative abundance
**
***
*
DMSO IACS DMSO IACS
EV
NDI1
K
Aspartate relative abundance
*
ns
ns
DMSO IACS DMSO IACS
EV
NDI1
L
Citrate relative abundance
**
*
ns
DMSO IACS DMSO IACS
EV
NDI1
M
Fold increase in SK-N-AS xenograft volume over 5 days
**
ns
DMSO
IACS
DMSO
IACS
EV
NDI1
N
Cell Viability (% of control)
0uM Palmitate-BSA
200uM Palmitate-BSA
Log IACS concentration (uM)
O
Cell count
p <0.0001
NS
DMSO -FA
IACS -FA
DMSO +FA
IACS +FA
IACS +FA +Eto

Figure S3: NDI1 expression rescues some IACS-010759-mediated metabolic alterations

(A - C) Untargeted metabolomics analysis demonstrates increased 3-hydroxyacylcarnitine abundance in IACS-010759 versus DMSO treated neuroblastoma (BE(2)C, A), melanoma (M481, B) and NSCLC (HCC827, C) xenografts. Red dots are 3-hydroxyacylcarnitines (BE(2)C DMSO n=7, IACS-010759 n=7; M481 DMSO n=5, IACS-010759 n=5; HCC827 DMSO n=4, IACS-010759 n=4).

(D) NAD^+/NADH ratio in DMSO- and IACS-010759-treated tumors. These ratios were determined by quantifying NAD^+ and NADH in each sample.

(E and F) Oxygen consumption and extracellular acidification rates in SK-N-AS cells expressing either NDI1 or a control vector (EV) and treated with either IACS-010759 or DMSO.

(G) Principal component analysis on metabolomics data from SK-N-AS tumors expressing NDI1 (DMSO n=6, IACS-010759 n=5) or EV control (DMSO n=5, IACS-010759 n=6).

(H - L) Relative abundance of TCA cycle-related metabolites from SK-N-AS tumors expressing either NDI1 or EV control (EV DMSO n=5, IACS-010759 n=6; NDI1 DMSO n=6, IACS-010759 n=5).

(M) Fold increase in SK-N-AS tumor volume over 5 days in tumors expressing NDI1 or EV control (EV DMSO n=5, IACS-010759 n=6; NDI1 DMSO n=6, IACS-010759 n=5).

(N) Viability of SK-N-AS cells co-treated with IACS-010759 and 200μM palmitate.

(O) SK-N-AS cell count following 72 hours incubation with 1μM IACS-010759, 100μM palmitate and 5μM etomoxir. Statistical significance was assessed using Student's *t*-test (D), one-way ANOVA followed by Sidak's multiple comparisons adjustment (E, F), Welch's one-way ANOVA test followed by Dunnett's T3 method (H-J, L, O) or Kruskal-Wallis test followed by Dunn's method (K) for multiple comparisons adjustment on the $\log_2$ transformed data, or $\log_2$-transformed one-way ANOVA followed by Sidak's multiple comparisons adjustment (M). Statistical tests were two-sided. Data represent mean ± SD. *p = 0.01 - 0.05, **p = 0.001 - 0.01, ***p = 0.0001 - 0.001, ****p < 0.0001.

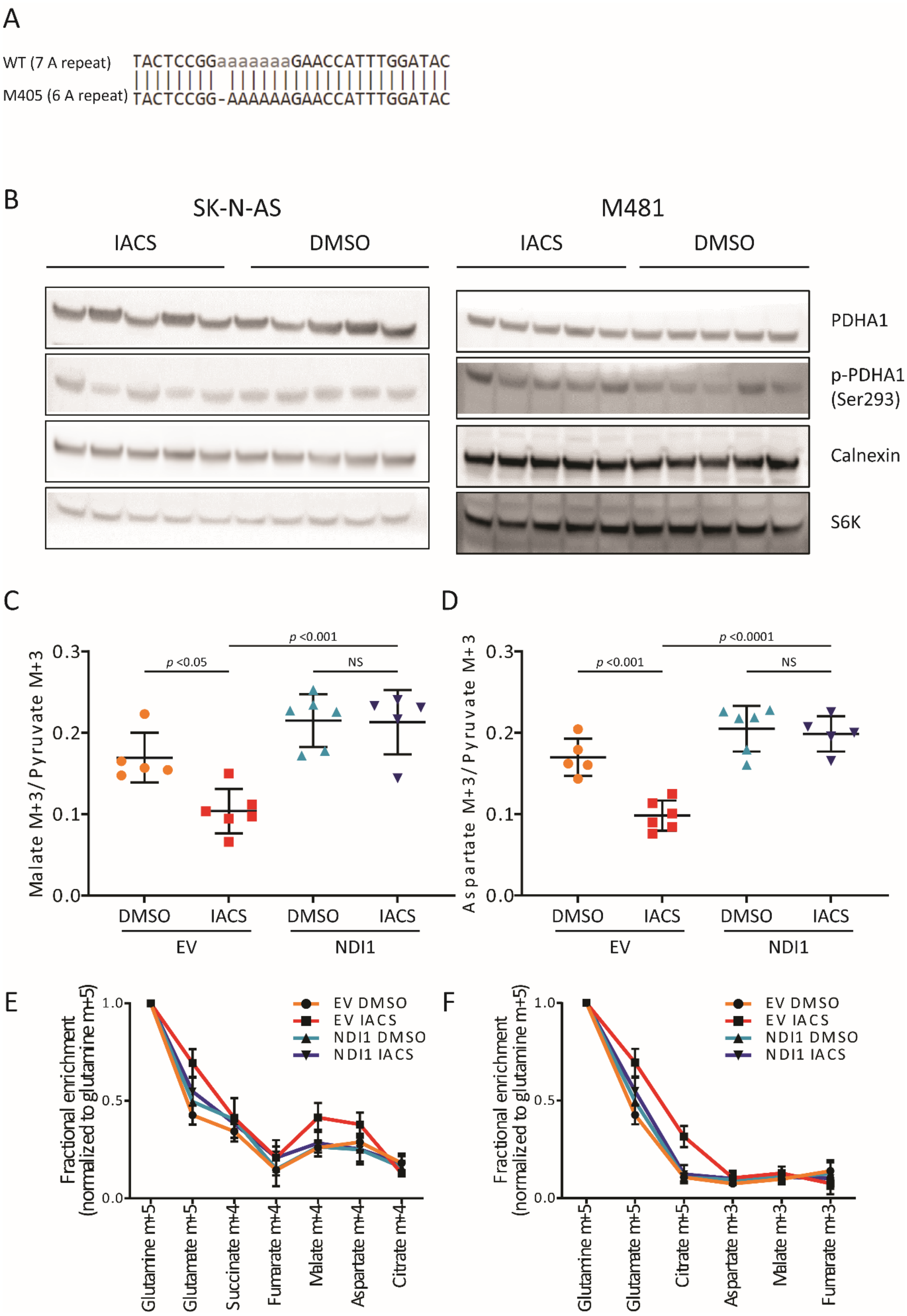
A
WT (7 A repeat) TACTCCGGaaaaaaaGAACCATTTGGATAC
M405 (6 A repeat) TACTCCGG-AAAAAAGAACCATTTGGATAC
B
SK-N-AS
M481
IACS
DMSO
PDHA1
p-PDHA1 (Ser293)
Calnexin
S6K
C
Malate M+3/ Pyruvate M+3
p <0.05
p <0.001
NS
DMSO IACS DMSO IACS
EV NDI1
D
Aspartate M+3/ Pyruvate M+3
p <0.001
p <0.0001
NS
E
Fractional enrichment (normalized to glutamine m+5)
EV DMSO
EV IACS
NDI1 DMSO
NDI1 IACS
Glutamine m+5
Glutamate m+5
Succinate m+4
Fumarate m+4
Malate m+4
Aspartate m+4
Citrate m+4
F
Citrate m+5
Aspartate m+3
Malate m+3
Fumarate m+3

Figure S4: Additional metabolic effects of IACS-010759 in xenografts

(A) Single nucleotide deletion in the *MT-CO1* gene detected in mitochondrial DNA (mtDNA) from M405 xenografts.

(B) Western blot showing PDHA1 and p-PDHA1 (Ser293) expression in DMSO and IACS-010759 treated SK-N-AS and M481 xenografts.

(C) Malate m+3/pyruvate m+3 ratio in SK-N-AS xenografts expressing EV or NDI1 and treated with DMSO or IACS-010759.

(D) Aspartate m+3/pyruvate m+3 ratio in SK-N-AS xenografts expressing EV or NDI1 and treated with DMSO or IACS-010759.

(E-F) Fractional enrichment in the oxidative (E) and reductive (F) pathways normalized to glutamine m+5 in SK-N-AS xenograft tumors expressing either NDI1 or a control vector (EV). Tumors were treated with either IACS-010759 or DMSO (EV DMSO n=6, IACS-010759 n=6; NDI1 DMSO n=7, IACS-010759 n=7).

Statistical significance was assessed using one-way ANOVA followed by Sidak's multiple comparisons adjustment (C-F). Statistical tests were two-sided. Data represent mean ± SD. *p = 0.01 - 0.05, **p = 0.001 - 0.01, ***p = 0.0001 - 0.001.
